# Boom Chack Boom—A multimethod investigation of motor inhibition in professional drummers

**DOI:** 10.1002/brb3.1490

**Published:** 2019-12-04

**Authors:** Lara Schlaffke, Sarah Friedrich, Martin Tegenthoff, Onur Güntürkün, Erhan Genç, Sebastian Ocklenburg

**Affiliations:** ^1^ Department of Neurology BG‐Kliniken Bergmannsheil Ruhr University Bochum Bochum Germany; ^2^ Institute of Cognitive Neuroscience, Biopsychology Department of Psychology Ruhr‐University Bochum Bochum Germany; ^3^ Department of Psychology University of Duisburg‐Essen Essen Germany

**Keywords:** cortical motor networks, drummer, DTI, GABA spectroscopy, motor decoupling

## Abstract

**Introduction:**

Our hands are the primary means for motor interaction with the environment, and their neural organization is fundamentally asymmetric: While most individuals can perform easy motor tasks with two hands equally well, only very few individuals can perform complex fine motor tasks with both hands at a similar level of performance. The reason why this phenomenon is so rare is not well understood. Professional drummers represent a unique population to study it, as they have remarkable abilities to perform complex motor tasks with their two limbs independently.

**Methods:**

Here, we used a multimethod neuroimaging approach to investigate the structural, functional, and biochemical correlates of fine motor behavior in professional drummers (*n* = 20) and nonmusical controls (*n* = 24).

**Results:**

Our results show that drummers have higher microstructural diffusion properties in the corpus callosum than controls. This parameter also predicts drumming performance and GABA levels in the motor cortex. Moreover, drummers show less activation in the motor cortex when performing a finger‐tapping task than controls.

**Conclusion:**

In conclusion, professional drumming is associated with a more efficient neuronal design of cortical motor areas as well as a stronger link between commissural structure and biochemical parameters associated with motor inhibition.

## Graphical abstract

Our multimethodal MRI data provide evidence that professional drumming is associated with a more efficient neuronal design of cortical motor areas, as well as a stronger link between commissural structure and biochemical parameters associated with motor inhibition. This shows that long‐term learning of complex motor tasks could lead to substantial restructuring in cortical motor networks, which could also have interesting implications for clinical applications for patients with motor disorders.

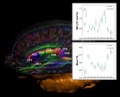

## 1 INTRODUCTION

Our hands are the primary means of interaction with the environment. A key aspect of hand use in humans is its asymmetrical organization. While most individuals can perform easy motor tasks with two hands at a similar level, only very few individuals can perform complex fine motor tasks with both hands equally well. Most individuals strongly prefer one hand (often called the dominant hand) over the other hand. Typically, each individual has a distinct handedness and prefers either the left or the right hand for complex fine motor tasks, for example writing (Güntürkün & Ocklenburg, 2017; Ocklenburg, Hugdahl, & Westerhausen, 2013). Handedness is thus one of the most pronounced and most widely investigated aspects of hemispheric asymmetries. A ratio of 90% right‐handed to 10% left‐handed people is constant for the past 5,000 years over all continents (Coren & Porac, 1977) and is noticeable even in utero (Hepper, Shahidullah, & White, 1990).

Each hand is controlled by the contralateral motor cortex. Neuronal correlates of handedness are mostly investigated by examining brain activity during more or less complex hand movement tasks. Such activities with the dominant hand are largely regulated by the contralateral hemisphere, whereas motor tasks with the nondominant hand are controlled more bilaterally by both hemispheres (van den Berg, Swinnen, & Wenderoth, 2011; Grabowska et al., 2012). The corpus callosum, as the major connecting pathway between hemispheres, was shown to have substantial influence on the characteristics of handedness (Hayashi et al., 2008; Westerhausen et al., 2004). Right‐handed people show a strong ipsilateral motor cortex de‐activation, when performing tasks with their dominant hand (Genç, Ocklenburg, Singer, & Güntürkün, 2015). In contrast, in left‐handed people, ipsilateral activations/de‐activation are equally pronounced, independent of the used hand. These findings demonstrate the correlation between ipsilateral activations and transcallosal inhibitions (Tzourio‐Mazoyer et al., 2015). Furthermore, patients with callosal agenesis, a hereditary condition in which the corpus callosum is absent in the brain, show a stronger tendency toward both‐handedness, for example not having a dominant hand (Ocklenburg, Ball, Wolf, Genç, & Güntürkün, 2015). Therefore, inhibitory functions of the corpus callosum represent an important aspect when understanding the neuronal correlates of handedness (Genç et al., 2015; Ocklenburg, Friedrich, Güntürkün, & Genç, 2016).

Since handedness can be partly altered through training (Perez et al., 2007), its constituent neural fundaments can change by learning. Neuroplasticity describes the adaption and cortical reorganization for example after training or learning a new skill. Functional plasticity of motor skills has been in the focus of neuroscientific research for decades. Already in the 1990s, it has been shown that playing the violin as a professional is influencing the somatosensory representations of the left (nondominant) hand (Elbert, Pantev, Wienbruch, Rockstroh, & Taub, 1995). Being able to play a music instrument on a professional level can also influence visuo‐motor (Buccino et al., 2004; Stewart et al., 2003; Vogt et al., 2007) as well as audio‐motor processes (Bangert et al., 2006; Baumann, Koeneke, Meyer, Lutz, & Jäncke, 2005; Baumann et al., 2007; Parsons, Sergent, Hodges, & Fox, 2005).

Up to now, musical training‐driven plasticity was primarily centered on changes of cortical gray matter. However, most musical instruments are played with both hands, increasing the demand for fast, precise and uncoupled movements of both hands. When playing piano, both hands are recruited in an equally demanding manner and sometimes with different rhythm*s*, whereas playing a stringed instrument requires distinct motor activities for the same rhythm. In contrast, when drumming, both hands and even legs have to perform similar motor tasks, however with distinct rhythms. Therefore, drummers are well suited as subjects for the investigation of structural correlates of transcallosal inhibition.

While it is very difficult for an untrained person to play a ¾ beat with one hand and a 4/4 beat with the other at the same time, this is an easy task for trained drummers. Research in split‐brain patients indicates that this remarkable ability to uncouple the motor trajectories of the two hands is likely related to inhibitory functions of the corpus callosum. Franz, Eliassen, Ivry, and Gazzaniga (1996) investigated bimanual movements in split‐brain patients and healthy controls and found that the controls showed deviations in the trajectories when the two hands performed movements with different spatial demands (Franz et al., 1996). In contrast, split‐brain patients did not produce spatial deviations. This suggests that movement interference in controls is mediated by the corpus callosum and that professional drummers likely show an experience‐dependent change in callosal structure and/or function that enables them to perform two different motor trajectories with the two hands at the same time. Thus, drumming requires neuroplasticity of whiter matter pathways. This is what we set out to study.

The structural, functional, and biochemical correlates of this remarkable ability of professional drummers are still completely unclear, but unraveling them would yield important insights into the general neuronal foundations of motoric decoupling. Therefore, the present study was aimed at investigating professional drummers for structural, functional, and biochemical differences to untrained controls, linked to transcallosal inhibition. To this end, we used a state‐of‐the‐art multimethod neuroimaging approach. We assessed the microstructure of the corpus callosum using DTI to reveal possible alterations of callosal anatomy between groups (Friedrich et al., 2017; Genç, Bergmann, Singer, & Kohler, 2011a; Genç, Bergmann, Tong, et al., 2011b; Westerhausen et al., 2004). Moreover, we assessed the biochemical correlates of GABA spectroscopy to test long‐term changes of inhibitory motor control (Stagg, 2014), as GABA levels in motor regions are highly associated with BOLD activations and motor learning. Specifically, lower GABA levels are associated with an increased degree of motor learning (Ziemann, Muellbacher, Hallett, & Cohen, 2001), while individuals with higher baseline levels of M1 GABA have slower reaction times and smaller task‐related signal changes (Stagg, Bachtiar, & Johansen‐Berg, 2011). Last, we also scanned participants using a fMRI finger‐tapping task to use a well‐established quantitative framework producing different behavioral complexities (Genç et al., 2015; Haaland, Elsinger, Mayer, Durgerian, & Rao, 2004). We assumed that drummers should show differences from nonmusical controls reflecting a more efficient neural organization on the structural, functional, and biochemical modality.

## 2 METHODS

This study was approved by the local ethics committee of the Medical Faculty of Ruhr‐University Bochum (No. 16‐5718). Written informed consent was obtained from all participants, prior to study enrollment. All participants were treated in accordance with the declaration of Helsinki.

### 2.1 Subjects

A total of 48 young adults (26 ± 4.5 years of age; range: 18–38) participated in the present study. All participants were male, with no diagnosis of neurological disorders. Due to known effects of the menstrual cycle on the GABA level (Harada, Kubo, Nose, Nishitani, & Matsuda, 2011) and small availability of professional female drummers, only male participants were included. Twenty‐four participants were professional drummers with experience of 17 years on average (±6 range 8–33 years) and weekly training workload of 10.5 hr (±8; range 1.5–30 hr). All drummers were active members of music bands and/or worked as drumming teachers. Four subjects of the drummer group were excluded from the data analysis due to massive motion artifacts during scanning, yielding to final comparison of 20 drummers to 24 controls. According to the Edinburgh Handedness inventory (Oldfield, 1971) two subjects in each group were left‐handers, one additional drummer did not show a distinct handedness. There was no significant difference in handedness between the groups (*p* = .541).

### 2.2 Drumming test

**Figure 1:**
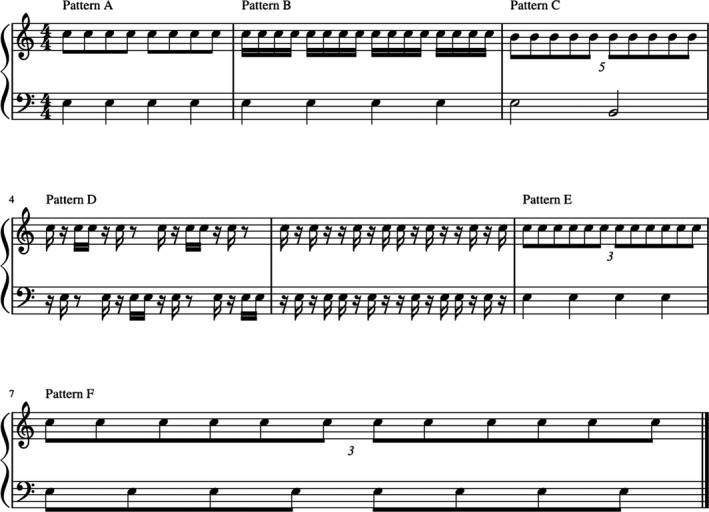
Schematic representation of the used drumming patterns with varying level of complexity

Prior to the MR‐examination, all participants had to perform a drumming test, using a Playstation 3 drumset and an edited version of Andrew Rudsons DrumBrain software (http://drumbrain.andrewrudson.com/). This software is using the Playstation 3 drum set as an electronic drum set, but unlike regular Playstation 3 music software it is not a game, but allows a realistic simulation of drum patterns. The test included six different drum rhythms with 120 bpm and varying levels of complexity (see Figure 1). These drum patterns were developed by the authors in consultation with a professional drummer and were piloted for complexity with two professional drummers not included in the test cohort. Additionally, two nonmusical controls also piloted the patterns to ensure that the patterns reliably reflect drumming skills. Participants scored, whenever they hit the correct drum within a 50 ms range of the predetermined marker set by the software. The maximum score (e.g., correct hits for all six patterns) was 1,088. The results of this test are shown as ratios between correct hits and the maximum score.

### 2.3 MR sequences

#### 2.3.1 Structural imaging

Magnetic resonance imaging (MRI) was performed on a 3T MR scanner (Philips Achieva 3T X) using a 32‐channel head coil. All participants were instructed to lay still throughout the complete scanning time, and the head was padded with cushions to reduce movement during scanning. Diffusion‐weighted imaging was performed using 60 diffusion‐weighted gradient directions (*b* = 1,000 s/mm^2^) with six interleaved non‐diffusion‐weighted image, yielding a total acquisition time of approximately ten minutes (FOV = 224 × 224 × 120 mm^3^, 2 × 2 × 2 mm^3^ voxel size, 60 slices, TR = 8,151 ms, TE = 88 ms). In addition, a high resolution MPRAGE T1‐weighted image was acquired as a reference for EPI‐distortion correction (FOV 256 × 256 × 220 mm^3^, 1 × 1 × 1 mm^3^ voxel size, TR = 8.3 ms, TE = 3.8 ms, 9° flip angle).

#### 2.3.2 GABA spectroscopy

The Mescher‐Garwood‐Point Resolved Spectroscopy (MEGA‐PRESS, Mescher, Merkle, Kirsch, Garwood, & Gruetter, 1998) sequence was used to obtain GABA‐edited spectra from single‐voxel acquisitions over the right and left sensorimotor cortices using the following parameters: voxel size 3 × 3 × 3 cm^3^, TR = 2,000 ms, TE = 80 ms, 20 ms sinc‐Gaussian editing pulses applied at 1.5 and 1.9 ppm (symmetric macromolecule suppression; MM‐s), 320 acquisitions in total with 20 averages of OFF and ON scans interleaved every 16 scans, spectral bandwidth of 2 kHz with a sampling rate of 2,048 points. Regional saturation technique slabs were applied to suppress fat signals from the skull. Interleaved water referencing (Edden et al., 2016) addressed B0 field offsets with prospective frequency correction using VAPOR. Specifically, for every 40 water‐suppressed acquisitions, a water‐unsuppressed acquisition was performed and used to correct the center frequency in real time. Thus, MM‐s GABA in this study refers to GABA excluding macromolecules. MRS sessions were scheduled so as to avoid effects of frequency drift on GABA‐edited MRS (Harris et al., 2014).

#### 2.3.3 fMRI task and sequence

During the task, T2*‐weighted echo‐planar images (single‐shot EPI) yielding 185 dynamic scans for the task were measured. The scan parameters were the following: FOV 224 × 224 mm^2^, 2 × 2 × 3 mm^3^ voxel size, 36 slices in an ascending scan order without gaps, TR = 2,500 ms, TE = 35 ms, 90° flip angle. The task included nine finger‐tapping conditions, which were represented visually using MR‐compatible LCD‐goggles (VisuaStim, Digital, Resonance Technology Inc.). The task was either to tap in moderate speed (approx. 2 Hz; on a MR‐compatible keyboard with the index finger, to tap a row index‐, middle‐, ring‐, small finger or a more complex tapping sequence (index‐, ring‐, middle‐, small finger) with either the right or the left or both hands simultaneously. Each of the nine conditions was presented two times for 25 s in a pseudorandomized order.

### 2.4 MR data analysis

#### 2.4.1 DTI data processing

All DTI processing steps were conducted using the ExploreDTI (Leemans, Jeurissen, Sijbers, & Jones, 2009) toolbox. Foremost, subject motion was corrected by rigid registration to the first acquired diffusion volume. Followed by corrections for distortions due to eddy currents and EPI deformations using nonrigid registration analysis. REKINDLE (Tax, Otte, Viergever, Dijkhuizen, & Leemans, 2015) was used to detect and remove outliers in combination with the weighted linear least squares estimation approach (Veraart, Sijbers, Sunaert, Leemans, & Jeurissen, 2013) to compute the diffusion tensor (Irfanoglu, Walker, Sarlls, Marenco, & Pierpaoli, 2012; Leemans & Jones, 2009). To correct for EPI distortions, the diffusion‐weighted images were nonrigidly aligned (image contrast during registration is the fractional anisotropy—FA) to the subjects' individual high resolution T1‐weighted image, with the deformation field constrained along the phase encoded A‐P axis. The ExploreDTI tool for atlas segmentation was used to extract the diffusion metrics from nine different corpus callosum segments along the A‐P axis according to the SRI24 atlas for normal human brains (https://www.nitrc.org/projects/sri24/). The atlas template was nonrigidly registered to each individuals’ space, and the atlas labels were respectively transformed. From the tensor the three eigenvectors *ν*_1–3_ and their scalar, the eigenvalues *λ*_1_−*λ*_3_ were calculated for each voxel. Fractional anisotropy (FA) and mean diffusivity (MD) were calculated based on the three eigenvalues using the following equations:

$$FA=\sqrt{\frac{1}{2}}\frac{\sqrt{{\left(\lambda_{1}-\lambda_{1}\right)}^{2}+{\left(\lambda_{2}-\lambda_{3}\right)}^{2}+{\left(\lambda_{3}-\lambda_{1}\right)}^{2}}}{\sqrt{\lambda_{1}^{2}+\lambda_{2}^{2}+\lambda_{3}^{2}}}$$

$$\text{MD}=\frac{\lambda_{1}+\lambda_{1}+\lambda_{3}}{3}$$

**Figure 2:**
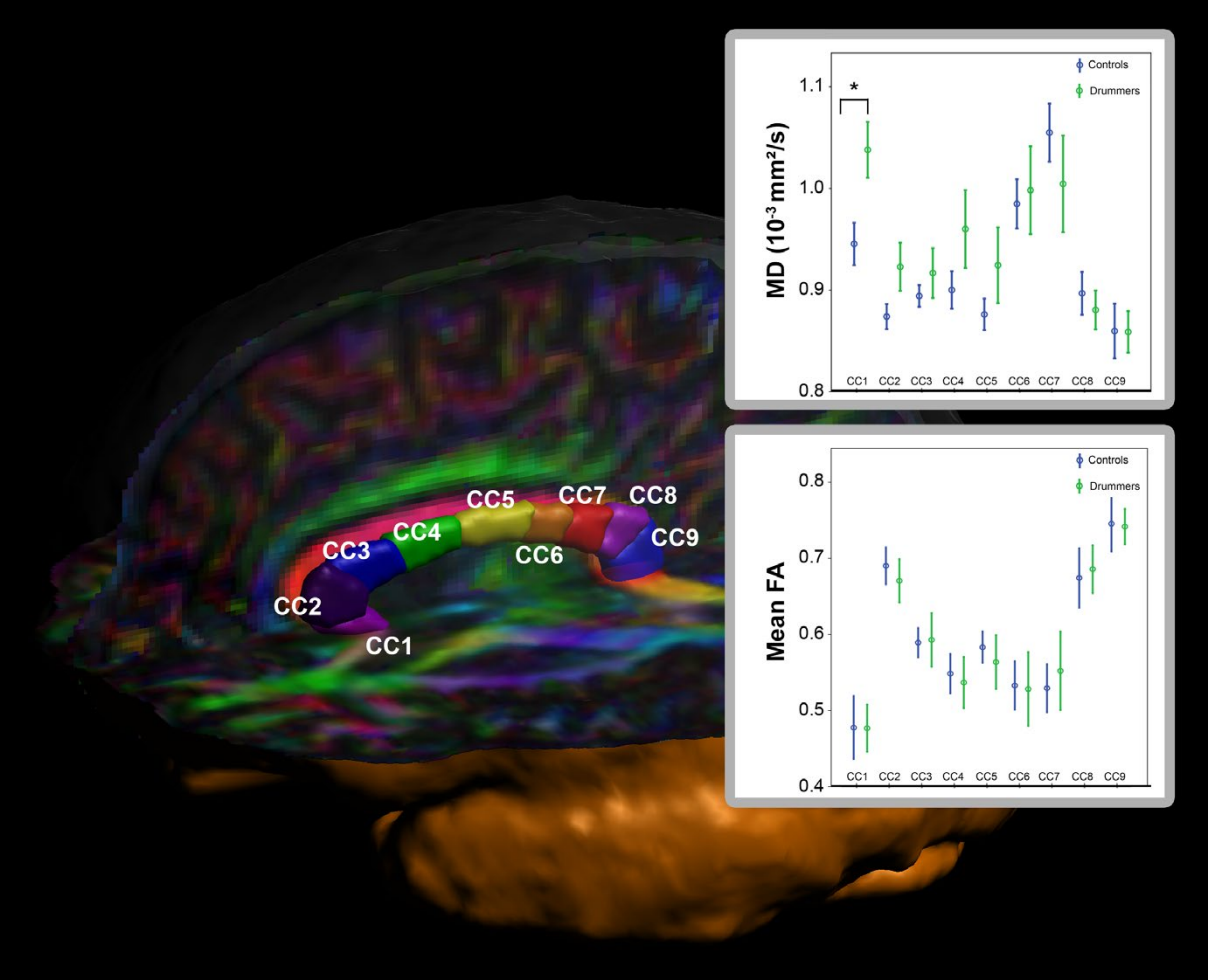
Segmentation of the corpus callosum into nine subsegments. The plot shows the mean MD values for drummers (green) and controls (blue) for all nine subsegments

Subsequently, the DTI‐metrics including FA and MD were estimated as means within the atlas labels. Figure 2 illustrates the corpus callosum segmentation of a representative individual.

#### 2.4.2 GABA spectroscopy analysis

GABA + concentration was calculated using the GABA Analysis Toolkit “Gannet,” which uses a Gaussian + baseline model to fit the edited GABA + signal and a Lorentz–Gaussian lineshape to fit the unsuppressed water signal (Edden et al., 2016). Brain volumes within each sensorimotor voxel (matching the MRS voxels) were segmented into gray matter (GM), white matter (WM), as well as cerebrospinal fluid (CSF) fractions using the segmentation routine implemented in the VBM8 toolbox. Institutional units for GABA+/H_2_O were corrected post hoc for voxel tissue fraction by calculating the ratio of GABA + units and the sum of GM and WM fractions according to the following equation: $\frac{\text{GABA concentration}}{\text{GM}\%+\text{WM}\%}$ × 100. They are stated as CSF‐corrected individual GABA + values.

#### 2.4.3 fMRI analysis

Functional images were converted from dicom to nifti and preprocessed using SPM8 (Welcome Department of Cognitive Neurology, University College) running under Matlab R2017b, (MathWorks). The preprocessing steps included realignment for movement correction, spatial normalization to the same stereo‐tactic space (using SPM EPI‐template) and spatial smoothing (FWHM: 6 mm). Within the first level analyses nine basic regressors, one for each condition (simple tapping, moderate tapping, difficult tapping with right, left or both hands), were modeled using a rectangular temporal profile (boxcar with a duration of a single block = 10 TRs) convolved with the hemodynamic response function. Variance estimations were performed within the general linear model (Friston et al., 1995), yielding nine b‐images, one per condition. The six movement parameters (rotation and translation) were added as nuisance variables to the model. In order to be able to explore complex three‐way or four‐way interactions in the data, we transferred fMRI data from SPM to SPSS by extracting the eigenvariates from a 10 mm sphere around the clusterpeak in the precentral gyrus. The cluster was derived by the contrast between the complex tapping task and the simple task (both hands) using a precentral gyrus mask (using the AAL atlas) with the coordinates [−36 | −14 | 64] in the left hemisphere, and [28 | −14 | 58] in the right hemisphere. The eigenvariates were subsequently transferred to SPSS for a 2 (Hemisphere: right, left) by 3 (hands used to tap: left, both, right) by 3 (task complexity: simple, moderate, difficult) by 2 (groups: drummers, controls) mixed repeated measures ANOVA.

## 3 RESULTS

### 3.1 Drumming performance

Professional drummers (overall drumming score = 83.06%, *SD*: 8.60) showed markedly better drumming performance than controls (62.58%, *SD*: 13.34). This effect reached significance (*t*_42_ = −5.91; *p* < .001, *d*_Cohen_ = −1.95).

### 3.2 Neuroanatomy of the corpus callosum

#### Fractional anisotropy

Individual FA values were analyzed using a 9 (corpus callosum segment: 1–9) by 2 (Group: Drummer or controls) mixed repeated measures ANOVA. The main effect of corpus callosum segment reached significance (*F*_8,42_ = 100.74; *p* < .001), indicating FA differences between the different subsegments of the corpus callosum, independently of group. In contrast, neither the interaction between corpus callosum segment and group (*p* = .64) nor the main effect of group (*p* = .88) reached significance.

#### Mean diffusivity

Individual MD values (see Figure 2) were analyzed using a 9 (corpus callosum) by 2 (Group: Drummer or controls) mixed repeated measures ANOVA. The main effect of corpus callosum segment reached significance (*F*_8,42_ = 17.55; *p* < .001, partial *η* ^2^ = 0.30), indicating MD differences between the different subsegments of the corpus callosum. Interestingly, this effect was modulated by group, as indicated by a significant interaction between corpus callosum subsegment and group (*F*_8,42_ = 2.49; *p* < .05, partial *η* ^2^ = 0.06). To further investigate this effect, post hoc *t* tests were used, revealing a significant difference in corpus callosum segment 1 (*p* = .009), with drummers having a higher MD (1.038 × 10^−3^ mm^2^/s) than controls (0.945 × 10^−3^ mm^2^/s). Moreover, we observed a nonsignificant trend in the same direction in segment 2 (*p* = .06). Although, the MD was also higher for the drummers in subsegment 2–6, all other segments failed to reach significance (all *p*‐values > .20). The main effect of group also failed to reach significance (*p* = .38). See boxes in Figure 2 for FA and MD values for both groups in all CC segments.

### 3.3 GABA spectroscopy

GABA spectroscopy data were analyzed using a 2 (hemisphere) by 2 (group) mixed repeated measure ANOVA. None of the effect reached significance (all *p*'s > .22).

### 3.4 Correlations between GABA spectroscopy and corpus callosum structure

**Figure 3:**
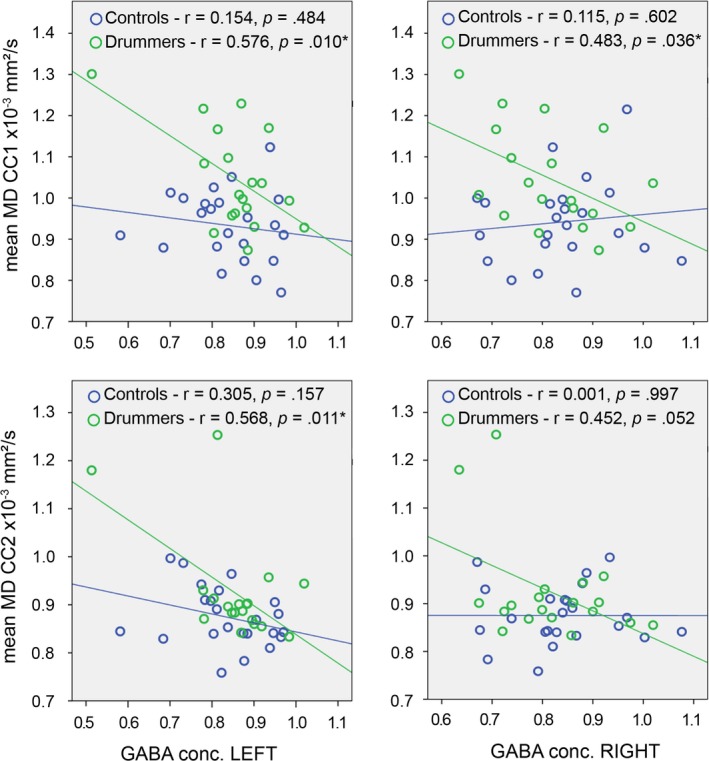
Correlations between GABA concentrations of the left and right GABA voxel and the MD of the first (top row) and second (bottom row) segment of the CC

In order to analyze, whether corpus callosum structure was relevant for GABAergic inhibition, we correlated the mean MD in corpus callosum segments 1 and 2 (the two segments that differentiated between the groups) with the GABA concentration in the left and right motor cortex (see Figure 3). Interestingly, we found substantial effects in professional drummers, but no statistically significant correlations in controls. In professional drummers, the GABA concentration in the left motor cortex correlated negatively with MD in both corpus callosum segment 1 (*r *= −.58; *p* = .010) and corpus callosum segment 2 (*r *= −.57; *p* = .011). The GABA concentration in the right motor cortex also correlated negatively with MD in both corpus callosum segment 1 (*r *= −.48; *p* = .036), but shortly failed to reach significance for corpus callosum segment 2 (*r *= −.45; *p* = .052). In controls, none of the correlations reached significance (all *p*‐values > .16).

#### 3.4.1 Prediction of behavioral performance by corpus callosum structure

**Figure 4:**
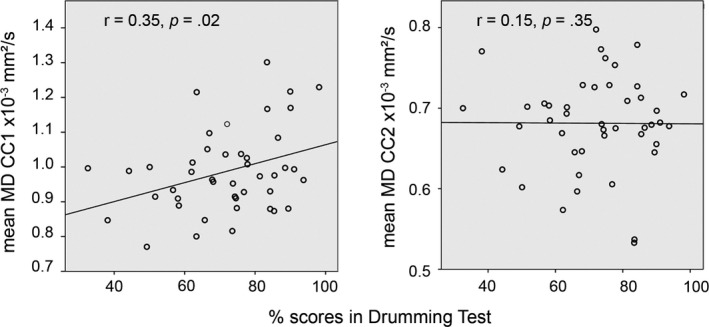
Correlations between drumming score and the MD in CC segment 1 (left) and CC segment 2 (right) in the professional drummers

Since mean MD in corpus callosum segments 1 and 2 differentiated between the groups, we correlated these measures with behavioral performance (drumming score) in order to investigate, whether interindividual variability of corpus callosum structure can predict differences in drumming proficiency. Here, we found a significant positive correlation (*r* = .35, *p* = .02) for corpus callosum segment 1, but not corpus callosum segment 2 (*r* = .15; *p* = .35), see Figure 4. This effect indicated that higher MD in corpus callosum segment 1 was linked to better drumming performance. No significant correlations were found for performance and GABA concentrations (GABA_left_: *p* = .82, GABA_right_: *p* = .40).

### 3.5 fMRI finger‐tapping task

#### 3.5.1 Eigenvariates of motor tapping

The eigenvariates describe the signal in a defined region compared to the whole brain mean signal. The fMRI data were analyzed using a 2 (Hemisphere: right, left) by 3 (hands used to tap: left, both, right) by 3 (task complexity: simple, moderate, difficult) by 2 (groups: drummers, controls) mixed repeated measures ANOVA. The main effect of group failed to reach significance (*p* = .84), but the main effects of hemisphere (*F*_1,42_ = 13.26; *p* = .001, partial *η* ^2^ = 0.24), hand (*F*_2,84_ = 163.28; *p* < .001, partial *η* ^2^ = 0.80), and task complexity (*F*_2,84_ = 163.11; *p* < .001, partial *η* ^2^ = 0.80) reached significance. These main effects, however, were modulated by several interactions. Among them hemisphere by hand (*F*_2,84_ = 292.60; *p* < .001, partial *η* ^2^ = 0.87), hemisphere by task complexity (*F*_2,84_ = 3.82; *p* < .05, partial *η* ^2^ = 0.08), hand by task complexity (*F*_4,168_ = 3.74; *p* < .01, partial *η* ^2^ = 0.08), and hemisphere by hand by task complexity (*F*_4,168_ = 85.86; *p* < .001, partial *η* ^2^ = 0.67).

Importantly, three interactions with group reached significance: task complexity by group (*F*_2,84_ = 8.07; *p* = .001, partial *η* ^2^ = 0.16), hand by task complexity by group (*F*_4,168_ = 3.19; *p* < .05, partial *η* ^2^ = 0.07), and hemisphere by hand by task complexity by group (*F*_4,168_ = 6.47; *p* < .001, partial *η* ^2^ = 0.13).

**Figure 5:**
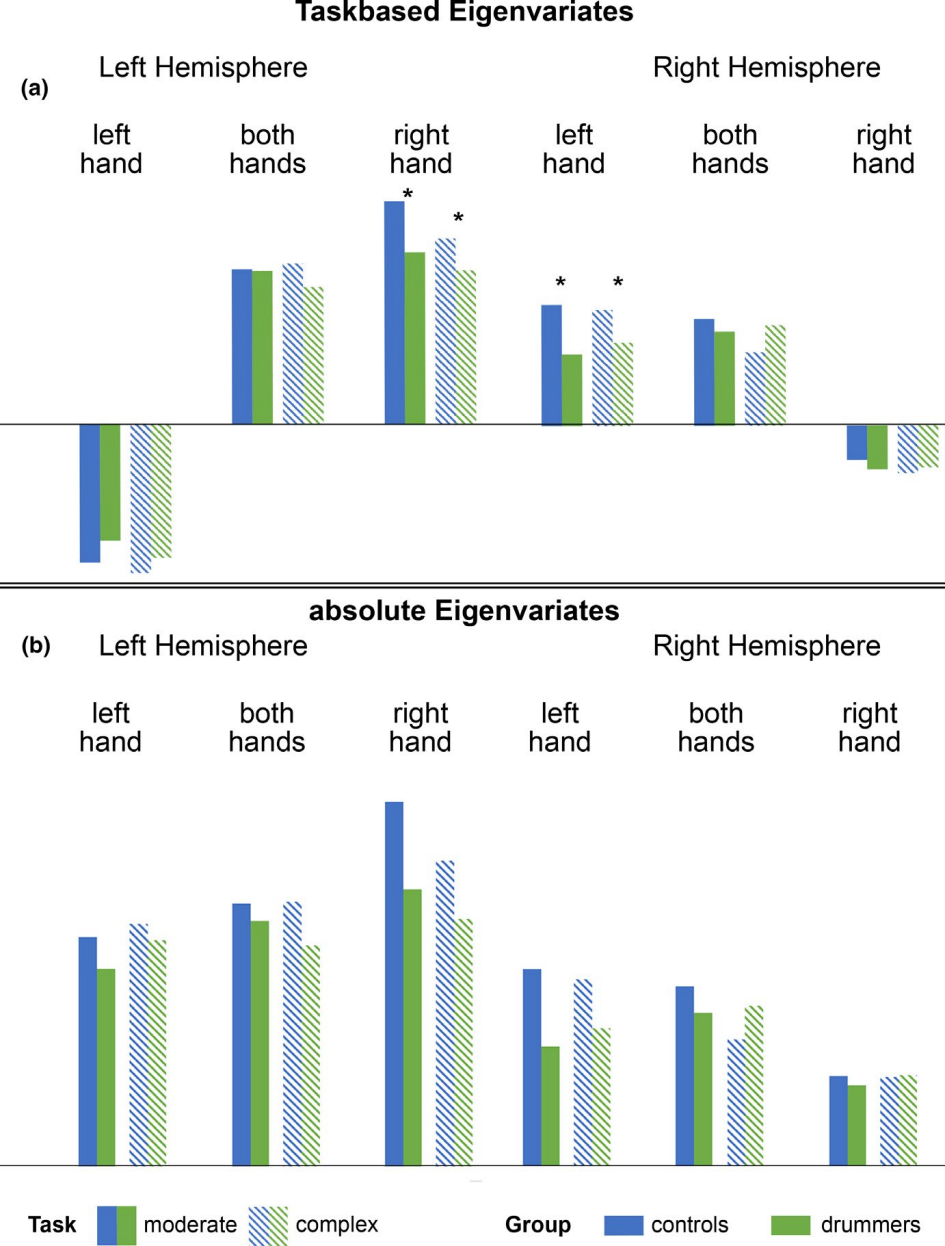
(a) Task‐based eigenvariates of a 10mm sphere in the motor cortex for both hemispheres, 3 task complexity levels and drummers and controls. (b) showing the absolute eigenvariates of the same sphere

As the hemisphere by hand by task complexity by group interaction was the highest order interaction that included the key factor group, we focused on further analyzing this interaction with post hoc tests (see also Figure 5a). None of the post hoc tests for the both hands condition reached significance (all *p*‐values > .10). For the right hemisphere, the group difference reached significance for all three conditions, when participants were tapping with their left hand (simple: *p* = .0007; moderate: *p* = .0004; difficult: *p* = .01), with drummers showing less activation/less de‐activation than controls.

Moreover, the group difference also reached significance for the simple condition for the right hemisphere, when participants were tapping with their right hands (*p* = .04). For the left hemisphere, the group difference reached significance for the moderate (*p* = .004) and the complex condition (*p* = .047) when participants were tapping with their right hands, while for the simple condition there was only a nonsignificant trend (*p* = .11). When participants were tapping with their left hand, the group comparison only reached significance for the simple condition *p* = .018) for the left hemisphere. All significant differences were driven by the drummers showing less activation/less de‐activation (see Figure 5a). Subsequent analysis of absolute values of the eigenvariates (see next section) point out that the absolute eigenvariates of the drummers were smaller than the controls (except for complex task, with both hands in the right hemisphere), see Figure 5b.

#### 3.5.2 Absolute eigenvariates of motor tapping

As the Eigenvalues can have both negative and positive values, a group difference for activation goes into another direction then a group difference for a de‐activation, making the integrated analysis of the data somewhat complicated (see Figure 5b). Therefore, we calculated the absolute eigenvariates in order to determine the degree of activation/de‐activation in the groups, independently of the direction of the effect. In order to determine, whether one group had a generally larger degree of activation, we calculated a 2 (hemisphere) by 2 (group) ANOVA. We found a significant main effect of group (*F*_1,42_ = 9.96; *p* = .003, partial *η* ^2^ = 0.19), showing that controls showed a generally larger degree of activation (2.08 ± 0.10) than drummers (1.60 ± 0.11). Moreover, the main effect of hemisphere reached significance (*F*_1,42_ = 84.20; *p* < .001, partial *η* ^2^ = 0.67), indicating a generally larger degree of activation in the left (2.35 ± 0.11) than in the right hemisphere (1.33 ± 0.07). The interaction failed to reach significance (*p* = .38).

## 4 DISCUSSION

The aim of the present study was to investigate the structural, functional, and biochemical correlates of fine motor behavior in professional drummers compared to nonmusical controls. This was done in order to understand the general principles underlying the ability for motoric decoupling between the hands—an ability that is at the core of drumming and similar musical activities. Besides testing drumming performance on the behavioral level, we assessed the microstructure of the corpus callous using DTI, the biochemical correlates of motor inhibition using GABA spectroscopy, and motor brain activation using an fMRI finger‐tapping task.

As expected, professional drummers showed a significantly better behavioral drumming performance than controls with a difference of more than 20% in scoring. This indicates that our drumming performance tests reliably differentiate professional drummers from nonmusical controls.

This enhanced motor performance in professional drummers was accompanied by structural differences in the neuroanatomy of the corpus callosum. Interestingly, we observed subsegment‐dependent MD differences between professional drummers and nonmusical controls. Drummers showed significantly higher MD in subsegment 1 of the corpus callosum than controls. Moreover, there was a nonsignificant trend for subsegment 2 and higher (not significant) MD in drummers in the subsegments 3–6, while there were no differences between groups for the other callosal subsegments (7–9).

Higher MD in the drummers reflects higher diffusion, indicating microstructural alterations. The changes associated with these cellular tissue properties cannot be uniquely identified (Alexander, Lee, Lazar, & Field, 2007). In the clinical context, higher MD has typically been related to lesion progression or white matter loss. For example, patients with multiple sclerosis show higher MD values in white matter structures than healthy controls (Kolasa et al., 2019). However, since we tested young and healthy adults, this interpretation does possibly not apply to our cohort. Here, it is conceivable that higher MD scores and thus more diffusivity could be related to fewer, but thicker fibers. Indeed, Westerhausen et al. (2006) demonstrated in healthy cohorts that callosal MD scores are linked to interhemispheric transfer time, a behavioral marker of callosal conduction speed. Specifically, the authors reported a negative correlation between MD and interhemispheric transfer time, indicating that higher MD in the CC is linked to faster transfer. Higher, however not significant, MD in the middle part of the CC (subsegments 3–6), where the motorfibers are located, would support the hypothesis, that structural differences in the CC may go along with better performance in motor inhibition. This may reach significance in a larger cohort. Since thicker fibers have faster conduction times, it is possible that the professional drummers of our sample had a more efficient (e.g., faster) anterior CC than the nonmusical controls. It is important to keep in mind that callosal space demands require that a relative increase of thicker fibers possibly goes along with a reduction of local fibers numbers (La Mantia & Rakic, 1990).

In line with the split‐brain literature (Franz et al., 1996), our data show that the corpus callosum is relevant for the ability to perform two different motor trajectories with the two hands. Specifically, our data show that the anterior corpus callosum connecting the frontal lobes is relevant for motor decoupling in professional drummers. Subsegments 1 and 2 of the corpus callosum in our study connect the orbitofrontal and prefrontal left and right cortices and are part of an area of the corpus callosum referred to as genu.

Interestingly, these segments of the corpus callosum are too anterior to directly reflect interhemispheric inhibition between the two primary motor cortices. However, it is known that the prefrontal cortex also plays a role in interhemispheric motor inhibition, particularly in relation to decision‐making. Ni et al. (2009) could show that interhemispheric motor inhibition consists of two different phases, the SIHI (short latency interhemispheric inhibition) and the LIHI (long latency interhemispheric inhibition). They could show that these inhibition processes are not limited to the direct connection of the left and right motor cortices, but rather use a widely distributed neuronal network related to different stages of motor control. These include the dorsolateral prefrontal cortex related to decision‐making during voluntary movement, as well as different areas related to motor planning and execution. Thus, our results suggest that within this network, particularly the prefrontal connections linking areas related to decision‐making play a crucial role for motor decoupling in professional drummers.

Regarding the biochemical differences between professional drummers and nonmusical controls, we did not observe any group differences in gray matter GABA concentration in the S1/M1 region. While we hypothesized that this region could differ between drummers and controls, we also did not observe any differences in the corpus callosum segment connecting these areas (subsegments 4/5). However, we found a strong and significant negative correlation between left and right M1 GABA concentration and corpus callosum MD in subsegments 1 and 2 in professional drummers, but not in controls. Thus, a more efficient anterior corpus callosum in drummers is linked to less GABA concentration in left and right M1. If, as outlined above, higher MD and therefore higher diffusivity indeed reflects thicker but fewer fibers in healthy participants, this relationship could be explained by our hypothesis that those drummers with the fewest fibers also have less GABAergic interneurons, but this relationship remains speculative. Future studies should use neurite imaging techniques like NODDI to specifically assess neurite density in drummers and healthy controls to test this assumption.

This relationship is particularly interesting given that the structurally different corpus callosum subsegments are not the ones connecting left and right M1. However, it can be assumed that due to years of training in professional drummers they did not only obtain a more efficient corpus callosum, but potentially also a tighter integration of the three subsequent components of motor inhibition according to Ni et al. (2009): voluntary movement decision‐making, motor planning, and motor execution. Thus, a more efficient connection between the decision‐making areas (involved in the decision whether to hit the drum or not at any given time point) could ultimately affect motor execution, which is also more efficient in this group indicated by the behavioral data.

This link was further substantiated by the fact that we observed a correlation between corpus callosum structure and drumming performance in drummers. In this group, higher MD in corpus callosum segment 1 was linked to better drumming performance. Thus, a more efficient anterior corpus callosum leads to better drumming performance.

In order to assess functional brain activation differences during motor performance between professional drummers and nonmusical controls, we used a finger‐tapping task (Witt, Laird, & Meyerand, 2008). For the eigenvariate analysis, we found several main effects and interactions, with the hemisphere by hand by task complexity by group interaction being the highest order interaction that reached significance. We therefore analyzed this interaction with Bonferroni‐corrected post hoc tests, which reached significance for several of the nine conditions for both the left and the right hemisphere. Importantly, the drummers showed less activation/ less de‐activation in all of these comparisons. In addition to the first analysis of fMRI, we also analyzed the absolute eigenvariates in order to determine, whether one group had a generally larger degree of activation than the other. This was done, as the Eigenvalues can have both negative and positive values, making it difficult to analyze group differences for activation and group difference for a de‐activation in the same analysis.

In line with the first analysis, professional drummers showed less activation/de‐activation in the motor cortex when performing a finger‐tapping task than controls in both hemispheres. This indicates that professional drumming is associated with a more efficient neuronal design of cortical motor areas. This finding is well in line with recent neuroscientific findings on so‐called sparse coding, for example having a more efficient neuronal architecture by using less neurons or smaller areas to execute a specific cognitive task (Genç et al., 2018). For example, in the area of intelligence research the so‐called neural efficiency hypothesis of intelligence has been brought forward, assuming that especially in rather simple tasks trained individuals show lower and therefore, more efficient brain activations (Genç et al., 2018; Neubauer & Fink, 2009).

Comparably, our data suggest that years of drumming training lead to a more efficient design of cortical motor networks, importantly including prefrontal networks. In addition to the main effect of group, our analysis of the absolute eigenvalues indicated a main effect of hemisphere, with more activation/de‐activation in the left then in the right hemisphere. This finding is most likely due to the fact that most participants were right‐handers.

In conclusion, professional drumming is associated with a more efficient neuronal design of cortical motor areas as well as a stronger link between commissural structure and biochemical parameters associated with motor inhibition. This shows that long‐term learning of complex motor tasks could lead to substantial restructuring in cortical motor networks, which could also have interesting implication for clinical applications for patients with motor disorders. Moreover, our findings implicated that motor decoupling between the two hemispheres as observed in professional drummers might rely not only on motor execution areas like M1, but also prefrontal areas involved in motor planning.

## CONFLICT OF INTEREST

None declared.

## Data Availability

The data that support the findings of this study are available from the corresponding author upon reasonable request.
